# Parametric Study of Girders with Sinusoidal Corrugated Web

**DOI:** 10.3390/ma17246079

**Published:** 2024-12-12

**Authors:** Krzysztof Śledziewski, Marcin Górecki, Jakub Gajewski, Michał Rogala

**Affiliations:** 1 Faculty of Civil Engineering and Architecture, Lublin University of Technology, Nadbystrzycka 40, 20-618 Lublin, Poland; m.gorecki@pollub.pl; 2 Faculty of Mechanical Engineering, Lublin University of Technology, ul. Nadbystrzycka 38, 20-618 Lublin, Poland; j.gajewski@pollub.pl (J.G.); m.rogala@pollub.pl (M.R.)

**Keywords:** parametric study, sinusoidal corrugated web, sinusoidal wave length, sinusoidal wave amplitude, critical capacity, shear capacity

## Abstract

Recently, steel girders with sinusoidal corrugations have become increasingly popular compared to those with traditional flat webs. This paper presents the second part of the research on the application of corrugated plates with different sinusoidal profiles as webs in girders. Parametric studies have been carried out in both linear and nonlinear domains, based on a representative numerical model developed and validated by experimental results. The research focused on the influence of the sinusoidal shape of the web on the shear capacity of the girders and the ultimate failure mode. The analyses were carried out using Abaqus software. Based on the results of the numerical analyses, it was concluded that increasing the wavelength of the sinusoidal wave decreases the ultimate shear capacity of the girders. This parameter also influences the failure mode. The results show that the wave amplitude has a small effect on the critical capacity. However, the amplitude influences the increase in the post-critical load and the size of the plastic zones located in the webs during the final phase of failure. With regard to the geometric parameters of the web, it was found that increasing the web thickness significantly improves the performance of the girders, while the web height has a negligible effect. It was also shown that the design guidelines in Eurocode 3 are very conservative in terms of estimating the shear buckling capacity of beams with sinusoidal corrugated webs and significantly underestimate the values.

## 1. Introduction

Plate girders have been used for many years primarily as load-bearing elements in bridges and industrial structures. In a typical cross-section, a plate girder consists of two parallel flanges and a web, which accounts for approximately 30–40% of the total weight. The economic design of beams typically requires the use of slender webs, that is elements with a small thickness relative to their height and length. Slender webs are susceptible to local buckling. To eliminate this phenomenon, the web thickness is increased or transverse and/or longitudinal stiffeners are used to ensure the necessary stability for full shear capacity at critical points [1].

**Figure 1:**
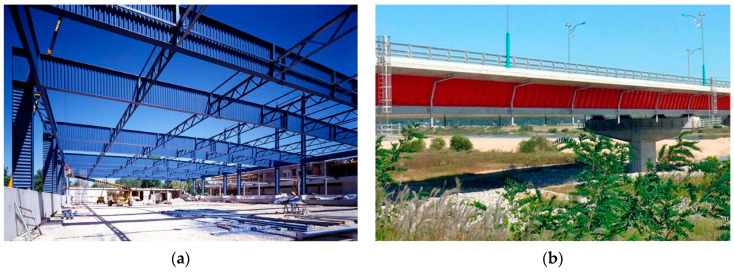
Application of corrugated webs in various types of engineering structures: (**a**) steel-framed hall [21]; (**b**) Ilsun Bridge [22].

An alternative to stiffeners is the use of corrugated sheet metal as a web. Several types of corrugations exist, including trapezoidal, triangular, channel-shaped, cellular, and sinusoidal [1]. Sinusoidal corrugated steel webs have gained popularity recently. They are primarily used in skeletal building structures [2] and bridge construction [3] (see Figure 1).

Previous research, regardless of the type of corrugation in the web [4,5,6,7], including that of the authors [8,9,10], has shown that the web contributes minimally to the transfer of normal stress. Studies [11,12,13,14,15,16,17] have demonstrated that the web is responsible for the shear capacity. Most experimental studies and numerical analyses have been conducted on webs made of trapezoidal corrugated sheets, owing to the ease of manufacturing such beams [18]. This situation differs for girders with sinusoidal webs produced using an automated process. The web sheet was cold-formed from hot-rolled flat sheets in coils and automatically welded to the flanges. A sinusoidal wave shape was formed using specially designed wave-rolling machines [19,20].

In the case of sinusoidal webs, previous experimental and parametric studies have primarily focused on the ultimate capacity and methods of estimation [23,24,25], failure propagation mechanisms owing to web buckling and the interactions between them [26,27,28], elastic buckling analyses of webs [29,30,31,32], flange buckling of girders [33,34,35], and the influence of shear on torsional buckling [30,36,37,38]. Recent studies have also examined the phenomena occurring in sinusoidal webs with openings [39,40,41]. In this case, practical applications outpaced the precise evaluation of the shear capacity of corrugated webs with openings. However, the number and scope of these studies on trapezoidal webs are limited. Given the continuous technological advancements and search for more economical solutions, it is necessary to conduct further research on the application of sinusoidal corrugated sheets as webs.

This paper presents the second part of our research on the application of corrugated sheets with various sinusoidal profiles as webs in steel girders. While experimental studies and an analysis of the results are presented in this article [42], this study focuses on parametric studies based on a representative numerical model. The influence of sinusoidal geometry on the shear capacity of girders and the final failure mode was investigated using Abaqus software 2023. Additionally, the obtained numerical values of the shear load limits were compared with the shear capacities calculated according to the guidelines in Appendix D of Eurocode 3 [43]. The symbols and notations used in this paper are shown in Appendix A.

## 2. Finite Element Models

**Figure 2:**
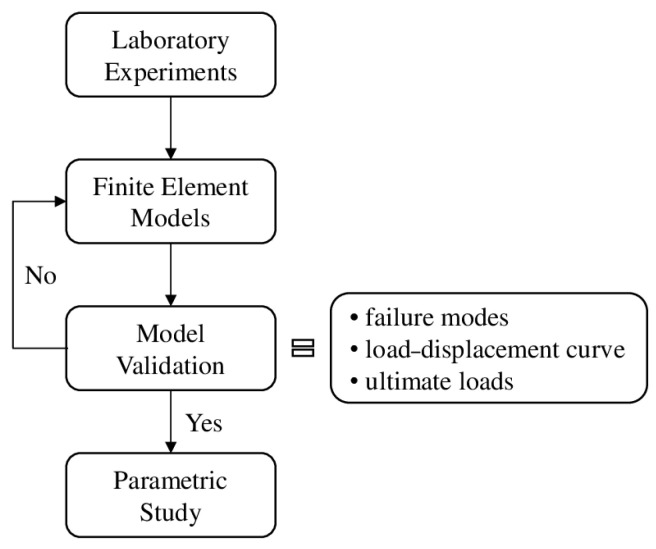
Research methodology.

The numerical analyses conducted were based on the destructive tests conducted on test specimens in a previous study [42]. The corresponding numerical models were developed using the ABAQUS finite element analysis software package (version 2023) [44], which allows for solving both linear and complex nonlinear tasks in engineering problems. The models were validated based on the results obtained from the laboratory experiments. Figure 2 presents the research methodology used in the form of a flowchart.

### 2.1. Description of Test Specimens

**Figure 3:**
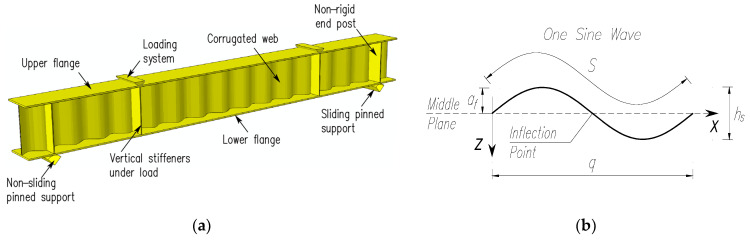
Test elements: (**a**) 3D model; (**b**) geometry of the web corrugation.

Figure 3a presents a 3D model view of the experimentally tested corrugated steel web girders, along with the geometry of a single web corrugation. The test specimens were designated as BS 155, BS 200, and BS 381.

The systems considered feature corrugated steel profiles with different sinusoidal waveforms. Table 1 summarizes the geometric parameters of the corrugated sheets used for the webs of the girders subjected to laboratory tests. The designations of the individual sinusoidal wave parameters are consistent with those in Figure 3b, where *a*_f_ represents the wave amplitude; *q* and *h*_s_ represent the wavelength projection and height, respectively; and *S* represents the developed wavelength.

**Figure 4:**
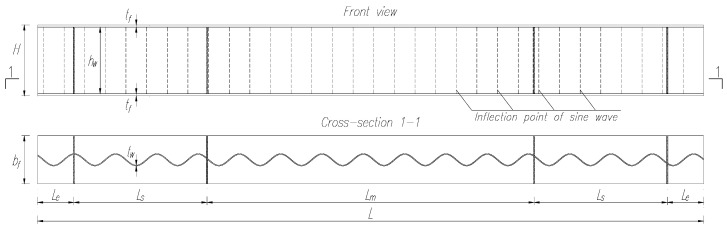
Geometry of beams tested experimentally.

The geometries of the tested girders and their designations are shown in Figure 4. The height (*H*) of the girders was 266 mm, including a web height (*h*_w_) of 250 mm and web thickness (*t*_w_) of 3 mm. The total length (*L*) was 2500 mm, consisting of a support zone segment length (*L*_s_) of 500 mm, a central segment length (*L*_m_) of 1228 mm, and the distance from the girder end to the support point (*L*_e_) of 136 mm. The lengths of the segments in the support zone were selected to maintain an *L*_s_/*h*_w_ of 2.0. The top and bottom flanges were made of flat steel sheets with a thickness (*t*_f_) of 8 mm and a width (*b*_f_) of 180 mm. At the support points and load application areas, the girders were reinforced with full-height double-sided vertical stiffeners made of flat steel plates with a thickness of 5 mm and variable width, depending on the corrugated sheet profile. The girders were connected using double-sided fillet welds with a thickness of 4 mm. The test specimens satisfied the criteria for low-rise girders.

### 2.2. Finite Element Type and Mesh

The experimentally tested girders were discretized using 3D shell elements with six degrees of freedom at each node, linear shape functions, and reduced integration (S4R).

**Figure 5:**
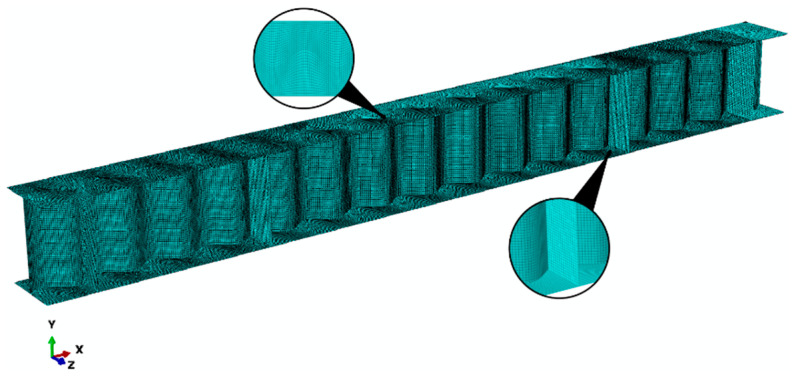
Generated finite element mesh of the shell model with a sinusoidal web.

Based on previous numerical studies [9], a global mesh size of ~5 mm was adopted. To ensure the interaction between the flanges, vertical stiffeners, and web shell (see Figure 5), manual modification of the mesh for individual components is necessary. Elements with aspect ratios of 1:1 were used for the web panel. The aspect ratios of the flange elements depended on their location. Near the edges of the flange, the elemental aspect ratio is 1:1, whereas in the central region, the flange element sizes are adjusted to match the web element dimensions. The vertical stiffeners featured elements of various lengths. Near the web, the elemental dimensions are maintained at an aspect ratio of 1:1. The remaining portions of the stiffeners were adjusted to the existing flange mesh, with an aspect ratio of approximately 1:2.

### 2.3. Material Properties

**Figure 6:**
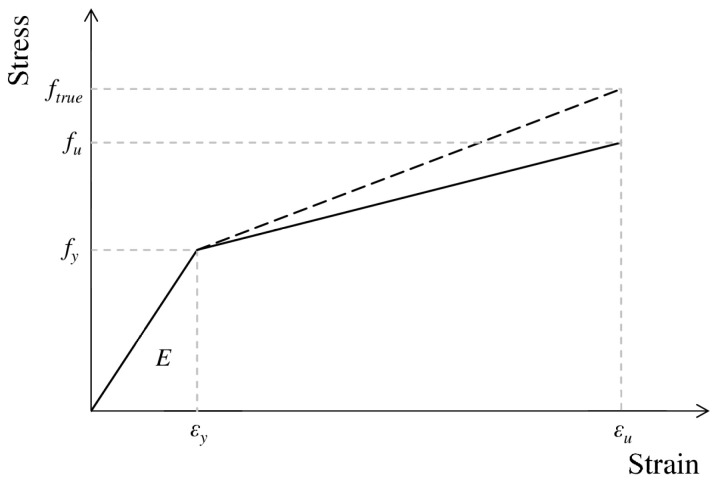
Nominal and true stress–strain characteristics of the steel.

The real stress–strain *ε-σ* behavior of the steel in the elastic–plastic range was used for the calculations, as shown in Figure 6. The material model was based on the results of the static tensile tests [45]. From the nominal stress–strain data (*ε*_nom_–σ_nom_), accounting for geometric and physical conditions, the true strain (*ε*_true_) was derived during specimen stretching, expressed in the natural logarithmic form:

(1) $$\varepsilon_{\mathrm{true}}=\ln\left(1+\varepsilon_{\mathrm{nom}}\right),$$

simultaneously, the true stress (*σ*_true_) was determined as follows:

(2) $$\sigma_{\mathrm{true}}=\sigma_{\mathrm{nom}}\left(1+\varepsilon_{\mathrm{nom}}\right).$$

Table 2 summarizes the material properties of individual girder components, including Young’s modulus (*E*), Poisson’s ratio (*v*), yield strength (*f*_y_), tensile strength (*f*_u_), and the corresponding ultimate strain (*ε*_u_).

### 2.4. Loading and Boundary Conditions

**Figure 7:**
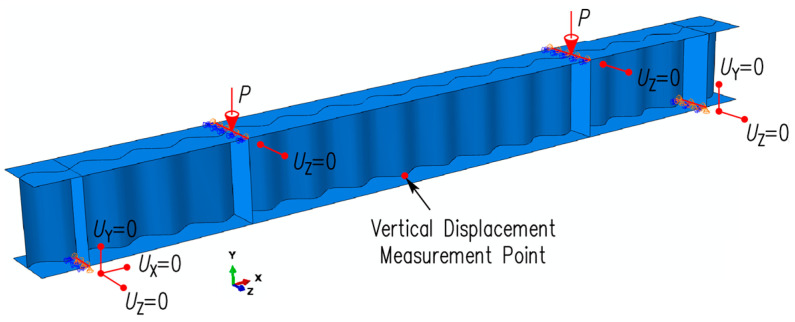
Boundary conditions and loading method for finite element models.

Figure 7 shows the finite element model of the girders under consideration. The boundary conditions assumed for the model replicated the experimental setup of four-point bending (see Figure 3a). The supports were defined linearly at the bottom flange and were precisely aligned with the axis of the double-sided stiffeners welded to the profile, eliminating the possibility of web deformation from the concentrated forces. On one support, displacements were restrained in the vertical (*U*_y_ = 0), longitudinal (*U*_x_ = 0), and lateral (*U*_z_ = 0) directions. At the second support, only the vertical (*U*_y_ = 0°) and lateral (*U*_z_ = 0°) displacements were restrained. To prevent lateral torsional buckling from influencing the failure mode of the girders, the lateral displacements (*U*_z_ = 0) were restricted to the locations of the intermediate vertical stiffeners. The load was applied as a pair of concentrated forces acting across the entire width of the top flange via the upper edges of the stiffening elements.

### 2.5. Applied Analysis and Geometric Imperfection

**Figure 8:**
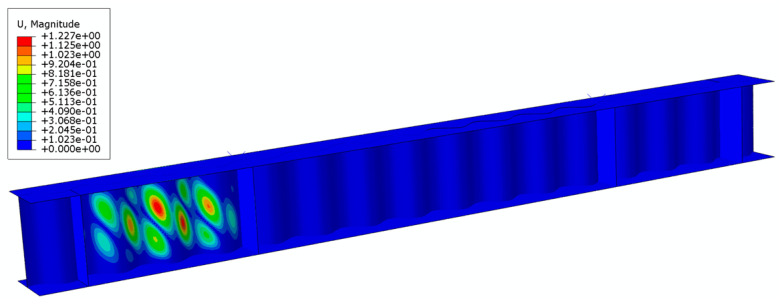
Typical first positive buckling mode of the corrugated web used as an imperfection shape of the girder.

To simulate the entire buckling process of the test specimens, geometrically and materially nonlinear analyses were performed, including imperfections. The elastic shear buckling load (*V*_cr_) and eigenmode shapes of the girders were obtained from a classical linear buckling analysis (the *BUCKLE procedure in ABAQUS). The first positive eigenmode (see Figure 8 for a typical example) was then used as the imperfect shape of the corrugated web girder. In the FEM analysis, the initial geometric imperfections were represented by an equivalent imperfection derived by scaling the maximum amplitude (I/A) to a value of *h*_w_/1000 according to previous measurements [14,24], where *h*_w_ is the height of the girder web. The nonlinear analysis was conducted using the modified RIKS method [44,46], which enables the tracing of the load–displacement curves beyond the peak load and into the post-buckling phase. The ultimate shear load (*V*_u_) was considered as the peak value of the shear load response of the test specimen.

### 2.6. Model Validation

**Figure 9:**
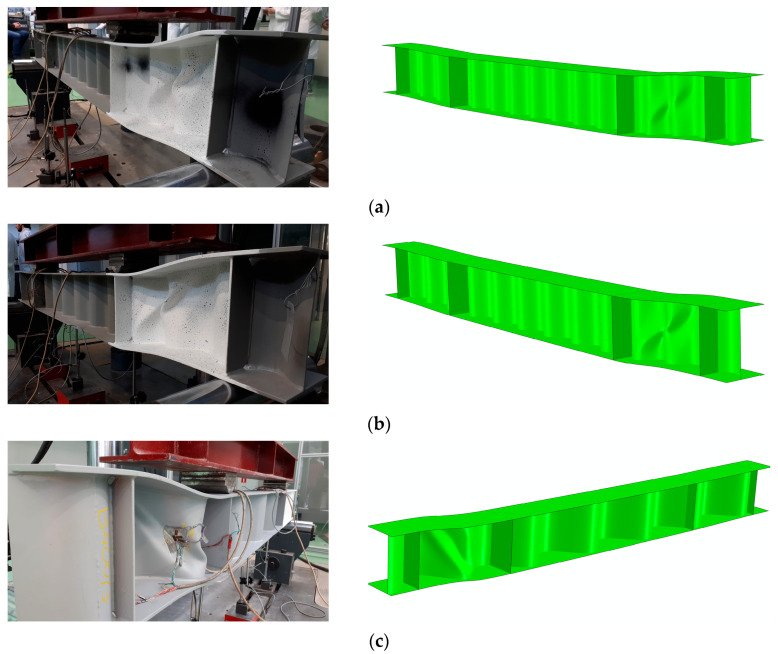
Comparison of the failure modes between test specimens and the numerical model: (**a**) BS 155, (**b**) BS 200, (**c**) BS 381.

Figure 9 compares the experimentally and numerically obtained failure modes for the three test specimens, indicating a close correlation in terms of the deformation patterns. Failure is caused by loss of web stability. Local web buckling began with localized buckling in the flat segments between the corrugations. These existing buckling zones led to the formation of plastic hinge lines, progressing towards the top flanges, and eventually causing the flanges to collapse in the plane of the girder owing to the sudden application of a transverse force. This phenomenon was similarly observed in the numerical simulations.

**Figure 10:**
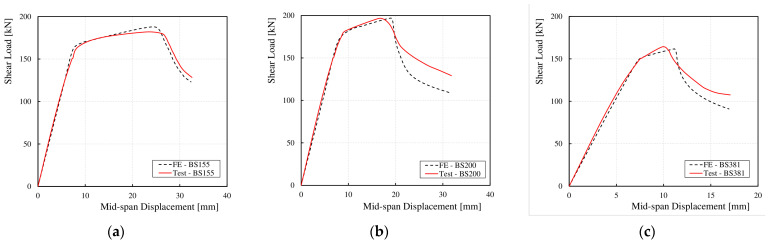
Load–deflection curves for the test specimens at mid-span: (**a**) BS 155, (**b**) BS 200, (**c**) BS 381.

The correlation between the shear force and vertical displacement at the mid-span obtained from both the numerical analyses and experimental tests is presented in Figure 10. A high degree of consistency was observed between the FEM numerical and experimental results for all cases, from the zero-load state until the onset of web instability. After web instability and until the ultimate load was reached, the scatter in the results increased. This was attributed to differences in displacement measurement methods between the laboratory and numerical studies. In the laboratory tests, the coordinates of the displacement measurement device’s contact point with the specimen at the end of the test did not match the coordinates at the beginning of the test. As the deformation increased in three dimensions, the change in the initial point coordinates also increased. In the numerical calculations, designating a specific measurement point at the start of the test allowed for consistent data retrieval from that point as it moved in space with global deformation.

The consistency of the results was also observed with respect to the attainment of the ultimate shear load (*V*_u_), as listed in Table 3. The differences between the values obtained from the numerical analyses, *V*_u,FE_, and the experimental tests, *V*_u,T_, did not exceed 4%, with an average ratio of *V*_u,FE_*/V*_u,T_ of 1.006 and a standard deviation of 0.024.

The comparative analyses demonstrated good agreement between the numerical and experimental results, as well as general consistency between the computational model and assumptions regarding the material strength hypotheses. A representative numerical model based on the experimental results is used for further parametric studies.

## 3. Parametric Study

Experimental studies have demonstrated that the shear capacity of I-beams with corrugated webs is closely related to the sinusoidal profile parameters. The numerical models of the girders developed based on the laboratory results enabled further analyses of a much larger group of such girders. The primary goal of the parametric study was to determine the influence of the length and amplitude of a single corrugation on the load-bearing capacity of these systems and the ultimate failure mode.

The geometric dimensions of the experimental girder, designated as BS 155 (see Table 1), including the geometry of the corrugated web, were adopted as the baseline for which further parametric models were developed. Finite element models (FEMs) were prepared using the following parameters of the baseline girder: length of a single corrugation (*q*), corrugation amplitude (*a*_f_), web height (*h*_w_), and web thickness (*t*_w_). The values of the girder parameters were within the following ranges:

1. *q* from 155 mm to 620 mm with increments of 77.5 mm;
2. *a*_f_ from 20 mm to 30 mm with increments of o 0.5 mm;
3. *h*_w_ from 250 mm to 500 mm with increments of 125 mm;
4. *t*_w_ from 2.0 mm to 3.0 mm with increments of 0.5 mm.

In this study, 21 numerical models of girders with different sinusoidal profiles (*q* and *a*_f_) were generated, in which the web thickness or height was subsequently modified. Finally, the results obtained from the 49 numerical models are presented and analyzed.

## 4. Results

For the analyzed numerical models, both the linear and nonlinear behaviors of the beams under advanced plastic deformation states were considered. Owing to the irreversible process of stability loss in the corrugated web, the remaining load-bearing capacity after exceeding the ultimate load could not be used for the operation of the structure [47]. However, from a safety perspective, it serves as a safeguard against collapse in the form of the so-called “plastic stop” [48].

Table 4 presents the results of the numerical studies along with the detailed geometric parameters of the considered webs. The term *V*_cr,FE_ denotes the shear load corresponding to the onset of stability loss in the corrugated web, signaling the beginning of a change in the geometric shape of the wave. The symbol *V*_u,FE_ represents the ultimate load corresponding to the beam’s failure condition, indicated by the end of plastic buckling formation. This value corresponds to the ultimate shear capacity of the beam. Additionally, based on the von Mises criterion in accordance with Equation (3), where *τ*_y_ is the yield strength in shear,

(3) $$V_{\mathrm{pl},R}=\tau_{y}h_{w}t_{w}=\frac{f_{y}}{\sqrt{3}}h_{w}t_{w}.$$

The upper limit of the plastic shear capacity for the considered webs (*V*_pl,R_) was estimated, which allowed for the determination of correction factors for the shear capacity of the numerically analyzed girders.

### 4.1. Effect of the Wavelength (q)

To investigate the effect of the wavelength (*q*) on the shear capacity, the beams were modeled with varying degrees of corrugation. In the numerical analyses, the web was modeled using seven different values of *q*. A constant increment in the base value of *q* (155 mm) by ~77.5 mm (0.5*q*_b_) was assumed, reaching a maximum of 620 mm (4.0*q*_b_), where the last value meets the low-frequency sinusoidal (LFS) criterion [49].

**Figure 11:**
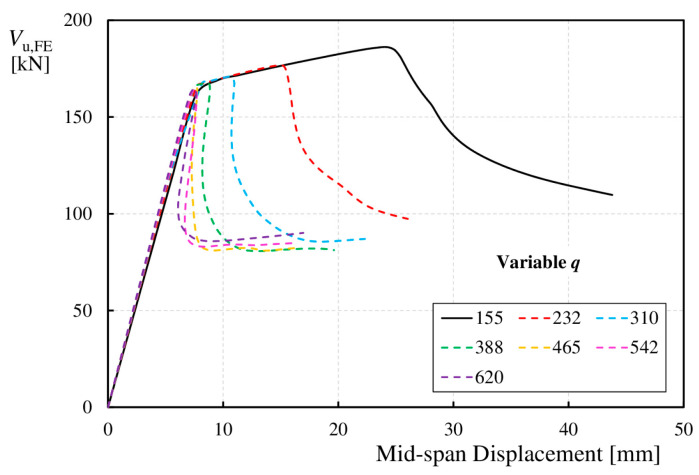
Relationship between load *V*_u,FE_ and vertical deflection at mid-span of the beam for Group 1 models.

Figure 11 shows the relationship between the maximum shear load and vertical displacement of the bottom flange at the mid-span of the tested elements for the Group 1 models. An increase in the fold pitch (*q*) caused a decrease in shear capacity (*V*_u,FE_). For beams with webs of lower corrugation (curves with *q* values ranging from 310 to 620 mm), after the ultimate capacity was exceeded, the shear force rapidly decreased, and the beam deflection decreased. In beams with webs having smaller fold pitches of 155 and 232 mm, after exceeding the ultimate capacity, the beams continued to deflect, and the shear force steadily decreased. Furthermore, an increase in *q* resulted in a reduction in the stiffness of the beams and the vertical deflection corresponding to the ultimate load (*V*_u,FE_), consequently reducing the post-critical load-bearing range (*V*_cr,FE_–*V*_u,FE_).

**Figure 12:**
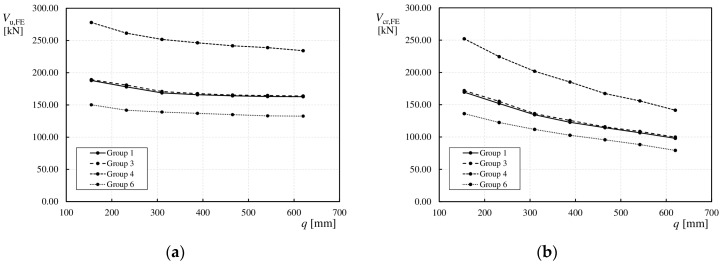
Relationships between load: (**a**) *V*_u,FE_ and corrugation length *q*; (**b**) *V*_cr,FE_ and corrugation length *q*.

In all the tested elements, both the ultimate load (*V*_u,FE_) and critical load (*V*_cr,FE_) decreased with increasing wavelength (*q*). This is a consistent trend (see Table 4), as confirmed by the relationships between *V*_u,FE_ and *V*_cr,FE_ as functions of *q*, as shown in Figure 12 for the selected groups of models.

From the relationship between *V*_u,FE_ and *q*, presented in Figure 12a, it can be seen that beams with webs with lower *q* values (155 and 232 mm) exhibited a larger decrease in the ultimate load (*V*_u,FE_) of approximately 5% compared to beams with larger q values. For fold lengths of 310 mm and above, the decrease was minimal, hovering at approximately 1%. The situation was different for the critical load (*V*_cr,FE_). In Figure 12b, where the relationship between *V*_cr,FE_ and *q* is shown, it is evident that the decrease is significant throughout the analyzed fold lengths, leading to a reduction in the elastic deformation range (0-*V*_cr,FE_). Initially, for smaller *q* values, the decrease is approximately 10%, after which it decreases to approximately 5% with an increasing fold length (*q*).

**Figure 13:**
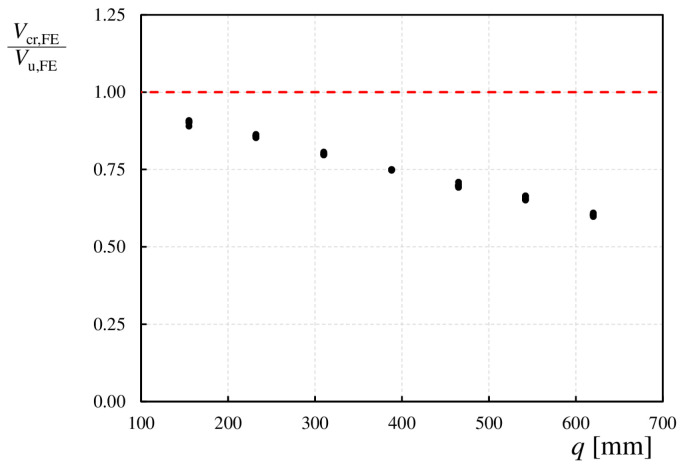
Comparison of the ratios of critical and ultimate loads for the tested models.

A detailed analysis of the load relationships shows that for the analyzed girders, the difference between the ultimate load (*V*_u,FE_) and critical load (*V*_cr,FE_) was initially relatively small, approximately 10% for *q* = 155 mm. As the fold pitch length (*q*) increases, the corrugated web approaches the shape of a flat web, and the difference between the loads *V*_cr,FE_ and *V*_u,FE_ increases, reaching up to 40% for *q* = 620 mm. This trend is noticeable for all the examined parameters, as shown in Figure 13.

**Figure 14:**
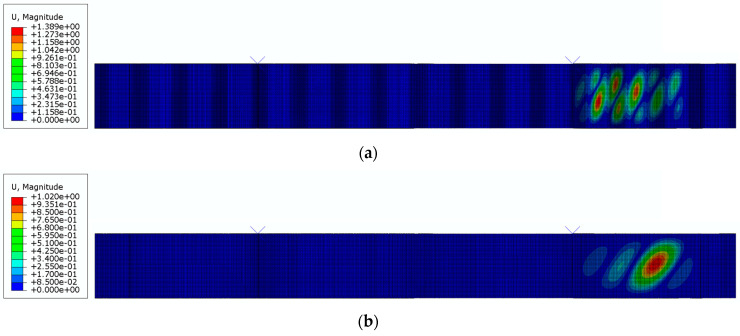
Example failure mechanisms of sinusoidal webs: (**a**) LB, (**b**) GB.

**Figure 15:**
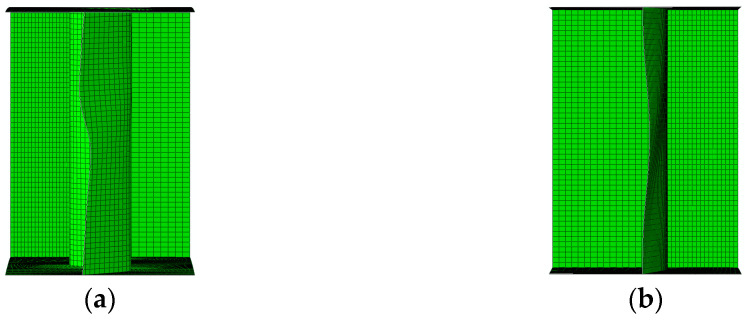
Final failure modes in cross-section: (**a**) LB, (**b**) GB.

Wavelength also affected the failure mode. In beams with corrugated webs, three failure modes can be identified: local, global, and interactive [50]. In beams with sinusoidally shaped webs, the dominant failure modes were local and global stability losses. However, as noted in [51], the interactive stability loss occurs differently than in trapezoidal webs. For the analyzed fold lengths (*q*), it was observed that with an increasing fold pitch, the failure mode of the web changed from local stability loss (for *q* values between 155 and 388 mm) to global stability loss (for *q* values between 465 and 620 mm). Figure 14 and Figure 15 show the typical buckling modes of the webs depending on the sinusoidal wavelength and the corresponding final failure patterns recorded during the numerical tests at the ultimate capacity. In the case of local buckling, damage occurs in the flat section between successive waves, whereas in the case of global damage, the buckling wave propagates diagonally across the web depth, connecting different folds in an arc shape.

It was also observed that, besides the failure mechanism, the location of the initiation points of stability loss changed. For *q* in the range of 155–388 mm, the stability loss process in the corrugated web begins with the formation of several local buckling focal points in the flat part of the wave near the rib. However, in the case of global web failure (for *q* values from 465 to 620 mm), owing to changes in the web stiffness, the buckling focal points shifted towards the edges of the beam. This phenomenon was observed in all the analyzed models subjected to parametric studies.

### 4.2. Effect of Wave Amplitude (a_f_)

The influence of wave amplitude (*a*_f_) on the shear capacity of girders with corrugated webs was studied based on a primary group of numerical models (Group 1). For each model, the amplitude was increased by 5 mm from 20 to 30 mm while keeping the wavelength constant.

From analyzing the results presented in Table 4 (Groups 1–3), it is clear that an increase in the depth of the corrugation (*a*_f_) results in a slight increase in the ultimate load value (*V*_u,FE_). A similar trend was observed for the critical load (*V*_cr,FE_). This increase was approximately 1% for *V*_u,FE_ and 2% for *V*_cr,FE_.

**Figure 16:**
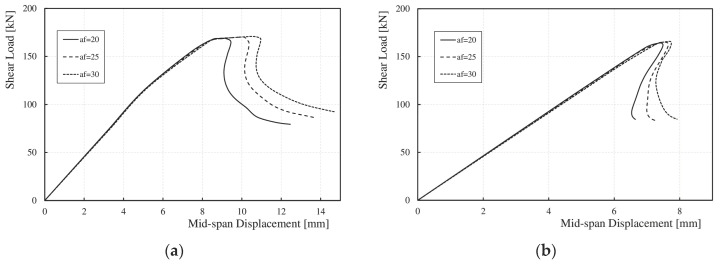
Relationship between load *V*_u,FE_ and vertical deflection at mid-span for corrugation lengths (**a**) 310 mm; (**b**) 465 mm.

Figure 16a,b show, for selected wavelengths (*q*), the relationships between the maximum shear load (*V*_u,FE_) and the vertical displacement of the bottom flange at the mid-span of the tested elements obtained from the numerical analyses. There was a noticeable increase in the post-critical capacity range (*V*_cr,FE_–*V*_u,FE_) owing to an increase in the deflection with a slight increase in the ultimate load (*V*_u,FE_). This phenomenon is a result of the accordion effect observed in corrugated webs [52,53]. The elastic deformation range remained unchanged in all the analyzed cases.

For girders with wavelengths (*q*) ranging from 155 to 310 mm, as the corrugation depth increased, the range of elastic–plastic deformations increased significantly by approximately 9–16%. For sinusoidal webs with *q* values between 388 and 620 mm, the increase was only between 1 and 5%.

For the girders analyzed in Groups 1–3, no change in the final failure mode was observed owing to the changes in wave amplitude. However, as the corrugation depth increases, the plasticity zones located on the webs expand. The deeper the corrugation, the larger the area of the web covered by the plasticity zone. This phenomenon was more pronounced in beams characterized by longer wavelengths, where the ratios of *V*_u,FE_/*V*_pl,R_ i.e., the ratio of the ultimate load to the plastic shear capacity, were lower and within the elastic range, as listed in Table 4.

**Figure 17:**
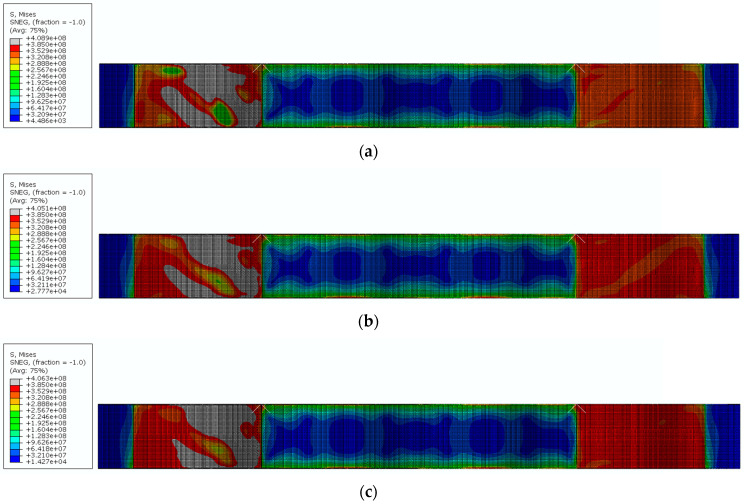
Failure mode and von Mises stress distribution for beams with a corrugation length of 542 mm depending on wave amplitude *a*_f_: (**a**) 20 mm, (**b**) 25 mm, (**c**) 30 mm.

Figure 17 illustrates the von Mises stress distribution for the final failure modes of models 6, 13, and 20 at the ultimate load. The red contours indicate the plastic regions, whereas the grey areas represent the locations exceeding the yield strength (*f*_y_). A noticeable increase in both the width and length of the plastic hinge zone along the diagonal lines of failure was observed.

### 4.3. Effect of Web Height (h_w_)

**Figure 18:**
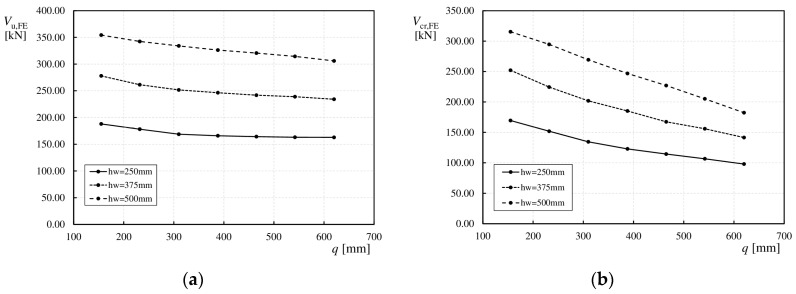
Influence of *h*_w_ on load values: (**a**) ultimate load *V*_u,FE_, (**b**) critical load *V*_cr,FE_.

Figure 18a and Figure 18b illustrate the effects of the web height (*h*_w_) on the ultimate (*V*_u,FE_) and critical load (*V*_cr,FE_) values, respectively. In this study, girders were modeled with three different web heights of 250, 375, and 500 mm, such that the ratios of *L*_s_/*h*_w_ (see Figure 3) were 1.0, 1.5, and 2.0, respectively.

It is widely known that *h*_w_ significantly affects the shear capacity of the girders. By analyzing Figure 18a and the numerical values presented in Table 4, it can be observed that for each wavelength, an increase in the web height resulted in a significant increase in the ultimate load values. As previously described, the trend is maintained that increasing wavelength (*q*) results in a decrease in shear load value. For web heights of 250 mm and 375 mm and wavelengths ranging from 155 mm to 310 mm, a sharper decrease in the capacity of approximately 5% was observed. For wavelengths (*q*) ranging from 388 mm to 620 mm, the decrease in capacity was negligible and steady at ~2%. In the case of girders with *h*_w_ = 500 mm, the decrease in the ultimate load was steady (almost linear) throughout the range of *q*. This is owing to the change in the failure mode from local to global for webs with denser corrugations. As shown in Figure 18b, in the case of the critical load (*V*_cr,FE_), an increase in web height led to an increase in the load values. However, the post-critical capacity decreases rapidly with an increase in the wavelength (*q*), particularly for girders with *h*_w_ = 500 mm.

**Figure 19:**
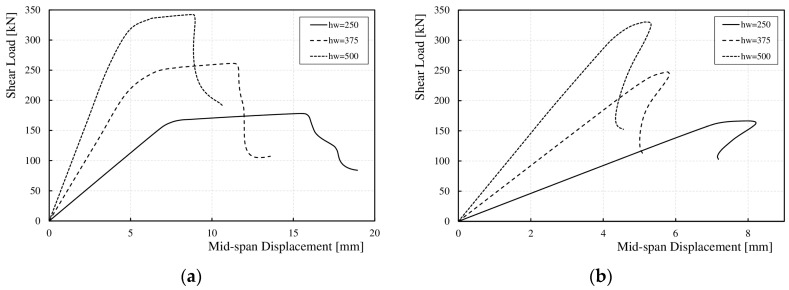
Influence of *h*_w_ on the relationship between ultimate load and vertical deflection at mid-span for corrugation lengths (**a**) 232 mm; (**b**) 388 mm.

For all the girders, as the web height increased, the range of elastic deformations (0-*V*_cr,FE_) also increased, whereas the range of post-critical capacity (*V*_cr,FE_–*V*_u,FE_) decreased, as shown for the selected models in Figure 19. It was observed that for girders with sinusoidal webs and wavelengths (*q*) of 155 and 232 mm, with heights (*h*_w_) of 375 and 500 mm, after exceeding the ultimate load (*V*_u,FE_), the load–deflection curve behavior changed. The shear force decreased rapidly without an increase in the deflection, as shown in Figure 19a. For other wavelengths (*q*), changing the web height (*h*_w_) did not affect the load–deflection curve, as shown in Figure 19b. It should be noted that an increase in web height increased the slenderness of the section.

Table 5 presents the influence of web height (*h*_w_) on the efficiency of the girders. The girders with a base wavelength of *q* = 155 mm were compared. Efficiency was determined by comparing the capacity per unit web height (*V*_u,*h*w_/*h*_w_) to the capacity for girders with a 250 mm web height ((*V*_u,*h*w_/*h*_w_)/(*V*_u,250_/250 mm)). Increasing the web height reduces the efficiency of the girders.

### 4.4. Effect of Web Thickness (t_w_)

To investigate the effect of web thickness (*t*_w_) on the performance of girders, girders from Group 1 were modeled with different thicknesses. Three values were considered: 2.0, 2.5, and 3.0 mm, which correspond to the most commonly used sinusoidal webs in girders for engineering structures [54].

**Figure 20:**
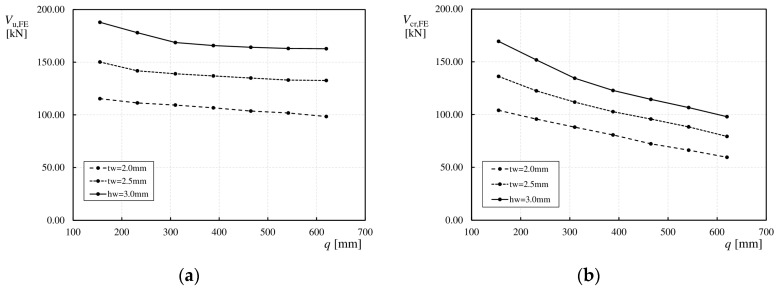
Influence of *t*_w_ on load values: (**a**) ultimate load *V*_u,FE_, (**b**) critical load *V*_cr,FE_.

The values of the ultimate load (*V*_u,FE_) and critical load (*V*_cr,FE_) with respect to the web thickness (*t*_w_) relative to the wavelength (*q*) are shown in Figure 20a,b. For girders, the web thickness (*t*_w_), such as the web height (*h*_w_), significantly affects the load-bearing capacity. As shown in the presented relationships, with an increase in the web thickness (*t*_w_), both the ultimate load (*V*_u,FE_) and critical load (*V*_cr,FE_) increased by approximately 20%.

These results confirm the earlier observation that with an increase in the wavelength (*q*), the load-bearing capacity decreased. For *t*_w_ = 2.0 mm, this decrease was linear owing to the uniformity of the final failure modes of the analyzed beams. However, for web thicknesses (*t*_w_) of 2.5 and 3.0 mm, the initial decrease was more rapid (approximately 5%) for larger corrugations, before it stabilized at approximately 2%.

**Figure 21:**
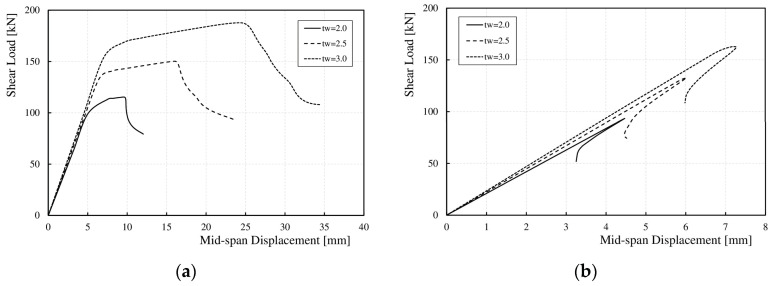
Influence of *t*_w_ on the relationship between ultimate load and vertical deflection at mid-span for corrugation lengths (**a**) 155 mm, (**b**) 620 mm.

Increasing the web thickness (*t*_w_) also resulted in an increase in the elastic deformation range (0–*V*_cr,FE_) and the elastic–plastic deformation range (*V*_cr,FE_–*V*_u,FE_). For *q* values between 155 and 310 mm, both ranges increased significantly, as shown in Figure 21a. For larger wavelengths (*q*) between 388 and 620 mm, a significant increase in the linear range was observed, with a slight increase in the nonlinear range, as shown in Figure 21b.

The impact of web thickness (*t*_w_) on the efficiency of girders was also analyzed by comparing the final load-bearing capacity per unit web thickness of the girder to the final load-bearing capacity per unit thickness for a girder with a web thickness of 2.0 mm (*V*_u,t*w*_/*t*_*w*_)/(*V*_u,2.0_/2.0 mm). The comparison was made for girders with a wavelength of *q* = 542 mm.

As shown in Table 6, increasing web thickness (*t*_w_) from *t*_w_ = 2.0 mm to *t*_w_ = 3.0 mm increases the efficiency of girders by up to 7%. This implies that the use of thick corrugated webs is more economical. Furthermore, the buckling capacity of a section under shear stress depends on the loaded area; an increase in thickness increases the area capable of carrying a buckling shear load. However, this capacity is limited by the yield strength.

### 4.5. Comparison of Calculated Design Shear Capacities According to Eurocode 3 with Results from Finite Element Analysis

Owing to the structural behavior of girders with corrugated webs, the interaction between the bending moments and shear forces can be disregarded when determining the shear capacity [55,56,57]. This assumption leads to the conclusion that the behavior of girders with corrugated webs can be compared to that of a truss system.

The guidelines for determining the shear capacity of such beams are provided in Annex D of the European Standard [43], which recommends determining the shear capacity of beams with sinusoidal corrugated webs based on local and global buckling. The equations proposed in the standard [43] were derived for a specific girder geometry [58]. Therefore, it is uncertain whether these equations can be applied to any general beam with a sinusoidal corrugated web, which is a concern raised by other researchers in their works [26,59].

Eurocode 3 recommends determining the shear capacity of girders with corrugated webs (*V*_Rd_) by using Equation (4).

(4) $$V_{\mathrm{Rd}}=V_{\mathrm{bw},\mathrm{Rd}}=\chi_{c}\frac{f_{y}}{\sqrt{3}}h_{w}t_{w},$$

where the shear buckling reduction factor (*χ*_c_) is equal to the smaller value of the local (*χ*_cL_) and global (*χ*_cG_) instability factors, which are calculated according to Equations (5) and (6).

(5) $$\chi_{cL}=\frac{1.15}{0.9+\lambda_{L}},$$

(6) $$\chi_{cG}=\frac{1.5}{0.5+\lambda_{G}}.$$

The reduction factors for the shear capacity were determined based on the web slenderness independently for local (7) and global (8) buckling considering critical stresses (9) and (10):

(7) $$5\lambda_{L}=\sqrt{\frac{f_{y}}{\tau_{crL}\sqrt{3}}},$$

(8) $$\tau_{crL}=\left(5.34+\frac{h_{s}s}{h_{w}t_{w}}\right)\frac{\pi^{2}E}{12(1-\nu^{2})}{\left(\frac{t_{w}}{s}\right)}^{2},$$

(9) $$\lambda_{G}=\sqrt{\frac{f_{y}}{\tau_{crG}\sqrt{3}}},$$

(10) $$\tau_{crG}=\frac{32.4}{t_{w}h_{w}^{2}}\sqrt[{4}]{D_{x}D_{y}^{3}},$$

where *E* is Young’s modulus, *υ* is Poisson’s ratio, and *D*_x_ and *D*_y_ are the plate bending stiffnesses in the plane perpendicular and parallel to the wave crest, respectively.

In general, it can be observed that in all parametric studies, the maximum shear forces obtained from the numerical analyses (*V*_u,FE_) are higher than the calculated shear capacities (*V*_Rd,EC_) determined according to the Eurocode 3 guidelines. As the wavelength increased, the difference also increased. For models with a base wavelength (*q*), the average ratio of *V*_u,FE_/*V*_Rd,EC3_ was 1.194 with a standard deviation of 0.013, whereas for a wavelength of *q* = 620 mm, the average value increased to 1.412 with a standard deviation of 0.047, as shown in Table 7, Table 8, Table 9, Table 10, Table 11, Table 12 and Table 13.

**Figure 22:**
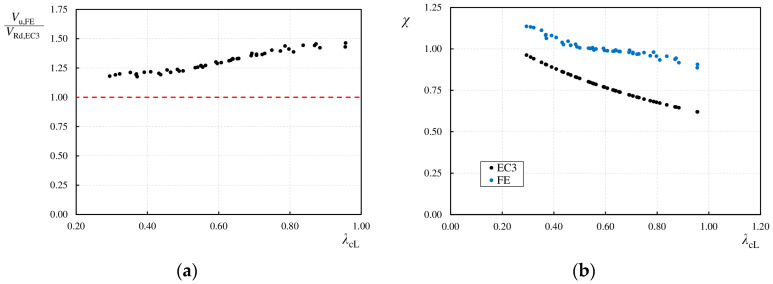
Comparison of analysis results: (**a**) ultimate load values; (**b**) buckling reduction factors and shear capacity correction factors for the numerically analyzed girders.

Figure 22a presents the relationship between the obtained results (*V*_u,FE_/*V*_Rd,EC3_) and the relative slenderness of the web plate (*λ_cL_*). As the slenderness (*λ_cL_*) of the web increases, there is a rapid rise in the ratio of numerically determined ultimate shear load to the calculated shear capacity (*V*_u,FE_/*V*_Rd,EC3_), reaching up to 40%. This is directly related to the method of estimating the shear capacity based on the critical stress values for local and global buckling, which are closely linked to the geometric shape of the web. Figure 22b shows the shear buckling reduction factors (*χ_c_*) determined according to Eurocode 3 [43], alongside the correction factors (*χ*_FE_ = *V*_u,FE_/*V*_pl,R_) derived from the numerical results. The correction factors (*χ*_FE_) are higher than the shear buckling factors (*χ_c_*). As the web plate slenderness increases, the difference between the correction factor (*χ*_FE_) and the standard buckling factor (*χ_c_*) grows.

This analysis indicates that the design guidelines in Eurocode 3 are highly conservative for estimating the shear buckling capacity of beams with sinusoidal corrugated webs. It is important to remember that these guidelines were developed based on the comparison of 70 experimental results and do not fully account for the variations in the geometry of sinusoidal webs. From a structural safety perspective, the calculated capacities are safe. However, this approach is not economical. For the base corrugation dimensions, for which the calculation procedures were developed, the shear capacities obtained from the numerical analyses were approximately 20% higher than those determined according to Eurocode 3.

### 4.6. Comparison of Calculated Design Shear Strength Resistance with Results from Finite Element Analysis

Eurocode 3 makes the shear strength dependent on the loss of global or local instability. This leads to the need to determine two independent critical strengths, (8) and (10). These parameters allow for the determination of relative slendernesses, which ultimately allow for the determination of the shear strength.

Sause and Braxton [60] suggested that the design shear strength resistance be determined from the following equation:

(11) $$\tau_{n,SB}=\tau_{y}{\left(\frac{1}{\lambda_{I,3}^{6}+2}\right)}^{1/3}.$$

Equation (11) was based on the interaction slenderness calculated according to (12) with a parameter size of n = 3. Local and global slenderness were determined from Equations (13) and (14):

(12) $$\lambda_{I,n}=\lambda_{L}\lambda_{G}{\left({\left(\frac{1}{\lambda_{L}}\right)}^{2n}+{\left(\frac{1}{\lambda_{G}}\right)}^{2n}\right)}^{1/2n},$$

(13) $$\lambda_{L}=\sqrt{\frac{12\left(1-v^{2}\right)\tau_{y}}{k_{L}\pi^{2}E}}\cdot \frac{w}{t_{w}},$$

(14) $$\lambda_{G}=\sqrt{\frac{12h_{w}^{2}\tau_{y}}{k_{G}F(\alpha ,\beta )Et_{w}^{0.5}b^{1.5}}}.$$

The global loss of the stability coefficient (*k*_G_) ranges from 26 to 68.4. In order to take into account the geometry of the web corrugation, the coefficient *F*(*α*_x_, *β*_x_) is determined:

(15) $$F(\alpha_{x},\beta_{x})=\sqrt{\frac{(1+\beta_{x})\sin^{3}\alpha_{x}}{\beta_{x}+\cos\alpha_{x}}}\cdot {\left(\frac{3\beta_{x}+1}{\beta_{x}^{2}(\beta_{x}+1)}\right)}^{0.75}.$$

This method deals with the determination of the design shear strength resistance for girders with trapezoidal webs. After approximating the sinusoidal shape, calculations can be performed for girders with sinusoidal webs.

The determination of shear strength resistance with interactive loss of stability has also been dealt with by Basiński [51], who proposed Formula (16):

(16) $$\tau_{n,BA}=\tau_{y}{\left(\frac{2}{\lambda_{I,6}^{6}+7}\right)}^{1/6},$$

where interactive slenderness (*λ*_I,6_) is dependent on interactive shear strength resistance (*τ*_crI,6_), as follows:

(17) $$\lambda_{I,6}=\sqrt{\frac{\tau_{y}}{\tau_{crI,6}}},$$

(18) $$\tau_{crI,6}=\frac{\tau_{cr,L}\cdot \tau_{cr,G}}{{\left(\tau_{cr,L}^{6}+\tau_{cr,G}^{6}\right)}^{1/6}}.$$

**Figure 23:**
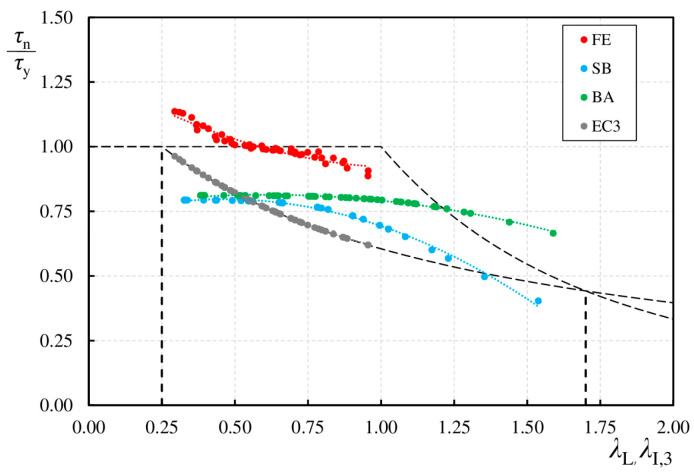
Normalized shear strength vs. slenderness.

Table 14 presents the collation of the results of design strength resistance obtained from numerical analysis (*τ*_FE_), proposed by Sause and Braxtan (*τ*_n,SB_), Basiński (*τ*_n,BA_), and EC 3 (*τ*_n,EC_). The normalized shear strength resistance obtained from the numerical analysis (*τ*_FE_/*τ*_y_) was also compared with the normalized resistance determined on the basis of the proposal of Sause and Braxtan (*τ*_n,SB_/*τ*_y_), Basiński (*τ*_n,BA_/*τ*_y_), and EC 3 (*τ*_n,EC_/*τ*_y_). Figure 23, on the other hand, shows a comparison of the normalized shear strengths versus slenderness.

By analyzing the results obtained and the distribution of slenderness, it can be seen that the Sause and Braxtan and Basiński models converge for corrugated beams with an interaction slenderness up to 0.5. In the Sause and Braxtan model, the ratio (τ_n,SB_/τ_y_) takes smaller values as the interaction slenderness increases. In addition, the calculated form of the normalized shear strength of the web according to Sause and Braxtan in the present comparison relates to the curve showing the local loss of stability. The curve based on the Basiński model is more similar to the curve representing the global loss of stability. The design shear strength versus slenderness obtained in the FE model has a similar character to the standard curve, indicating a local loss of stability.

This analysis indicates that other researchers’ proposals are also very conservative for the parameters studied in this article. Given the search for more economical solutions, further research is needed to develop universal models that can be applied to sinusoidal corrugated webs with different geometric parameters.

## 5. Conclusions

In this study, the influence of sinusoidal web profile parameters on the shear capacity and final failure mode of girders was investigated. Parametric studies were conducted based on a representative numerical model that was developed and validated using experimental results [42]. Based on detailed numerical investigations, the following conclusions were drawn:

1. For the analyzed geometrical conditions, an increase in the wavelength (*q*) led to a decrease in the shear capacity of the girder. Initially, the reduction in ultimate load (*V*_u_) is pronounced, but it stabilizes as the wavelength increases. In the case of critical load (*V*_cr_), there is a steady decrease throughout the analyzed wavelength range (*q*). The change in wavelength (*q*) also significantly affects the failure mode, shifting from local buckling to global buckling with an increase in damage initiation points.
2. For the investigated systems, the change in wave amplitude (*a*_f_) slightly increases their shear capacity (*V*_u_). It extends the post-critical capacity range (*V*_cr_–*V*_u_) and expands the plasticity zones localized at the web during the final failure stage.
3. A significant improvement in the shear capacity of the girder was observed with an increase in the web height (*h*_w_), while simultaneously reducing its efficiency. For all the analyzed girders, there was a significant increase in critical capacity (*V*_cr_), while the post-critical capacity range (*V*_cr_–*V*_u_) decreased. Additionally, increasing the height (*h*_w_) for girders with more corrugated webs caused a sharp decline in capacity after reaching the ultimate limit.
4. An increase in web thickness (*t*_w_) enhances both the critical capacity (*V*_cr_) and the ultimate shear capacity (*V*_u_) of girders. Furthermore, for girders with shorter wavelengths (*q*), a significant increase in the post-critical range *V*_cr_–*V*_u_ was observed. The efficiency of girders was also found to increase with an increase in web thickness (*t*_w_).
5. A comparison of the finite element analysis results with the Eurocode design guidelines for the parametric study cases shows that the Eurocode 3 design guidelines are highly conservative in estimating the shear capacity considering the buckling of beams with sinusoidal corrugated webs, significantly underestimating their values.

## Figures and Tables

**Table 1:** Geometric parameters of experimentally tested webs.

Test Element	*q*	*a_f_*	*h*_s_	*S*
	[mm]	[mm]	[mm]	[mm]
BS 155	155	20.0	40	181
BS 200	200	23.5	50	228
BS 381	381	68.5	140	487

**Table 2:** Results of the static tensile test of the steel.

Beam Element	*E*	*v*	*f*_y_	*f*_u_	*ε*_u_	*f*_u_ */f*_y_
	[GPa]	[-]	[MPa]	[MPa]	[%]	[-]
BS 155 web	204.97	0.34	384.45	488.56	23.55	1.14
BS 200 web	199.87	0.31	411.50	514.40	21.05	1.25
BS 381 web	206.89	0.31	347.92	476.68	27.47	1.37
Flanges/Stiffeners	181.77	0.27	367.46	498.35	23.73	1.36

**Table 3:** Comparison of ultimate loads from numerical and experimental results.

Test Element	*V*_u,FE_	*V*_u,T_	*V*_u,FE_/*V*_u,T_
	[kN]	[kN]	[-]
BS 155	187.93	181.80	1.034
BS 200	197.00	196.51	1.003
BS 381	161.80	164.17	0.986
Average value			1.007
Standard deviation			0.024

**Table 4:** Geometric parameters of the sinusoidal web considered, along with the numerical test results.

Variables	Model Group	Nr	*q*	*a*_f_	*h*_s_	*S*	*h*_w_	*t*_w_	*V*_pl,R_	*V*_cr,FE_	*V*_u,FE_
			[mm]	[mm]	[mm]	[mm]	[mm]	[mm]	[kN]	[kN]	[kN]
Variation in q	Group 1 (basic)	1	155	20	40	181	250	3.0	166.47	169.41	187.93
		2	232			251				151.82	178.05
		3	310			324				134.45	168.70
		4	388			400				124.82	166.43
		5	465			475				114.40	164.19
		6	542			550				106.61	163.07
		7	620			627				97.96	162.81
Variation in *a*_f_-*q*	Group 2	8	155	25	50	189	250	3.0	166.47	170.39	188.60
		9	232			257				153.57	179.95
		10	310			329				136.24	170.10
		11	388			403				125.23	167.23
		12	465			478				115.47	164.98
		13	542			553				107.11	163.95
		14	620			630				98.13	162.99
	Group 3	15	155	30	60	202	250	3.0	166.47	171.85	189.18
		16	232			267				155.16	180.81
		17	310			337				136.19	170.83
		18	388			410				125.58	167.57
		19	465			484				115.78	165.36
		20	542			558				108.61	164.66
		21	620			634				99.84	163.83
Variation in *h*_w_-*q*	Group 4	22	155	20	40	181	375	3.0	249.71	252.08	277.89
		23	232			251				224.44	261.34
		24	310			324				201.79	251.64
		25	388			400				185.14	247.37
		26	465			475				167.30	241.76
		27	542			550				158.86	238.90
		28	620			627				141.47	234.15
	Group 5	29	155	20	40	181	500	3.0	332.94	315.40	354.30
		30	232			251				294.65	342.33
		31	310			324				269.37	334.05
		32	388			400				246.77	330.22
		33	465			475				226.99	326.43
		34	542			550				205.10	314.37
		35	620			627				182.26	301.96
Variation in t_w_*-q*	Group 6	36	155	20	40	181	250	2.5	138.73	136.18	150.20
		37	232			251				122.47	141.84
		38	310			324				111.78	139.00
		39	388			400				102.70	136.97
		40	465			475				95.70	134.96
		41	542			550				88.28	133.04
		42	620			627				79.27	132.64
	Group 7	43	155	20	40	181	250	2.0	110.98	103.99	115.36
		44	232			251				95.65	111.31
		45	310			324				88.04	110.28
		46	388			400				80.67	107.66
		47	465			475				72.25	103.59
		48	542			550				66.25	101.77
		49	620			627				59.53	98.40

**Table 5:** Influence of web height on the efficiency of girders.

Model No.	*h*_w_	*V*_u,hw_	*V*_u,hw_/*h*_w_	$\frac{V_{u,\mathrm{hw}}{/h}_{w}}{V_{u,250}/250\;\mathrm{mm}}$
	[mm]	[kN]	[kN/mm]	[-]
1	250	187.93	0.752	1.00
22	375	277.89	0.741	0.99
29	500	354.30	0.709	0.94

**Table 6:** Influence of web thickness on the efficiency of girders.

Model No.	*t*_w_	*V*_u,tw_	*V*_u,tw_/*t*_w_	$\frac{V_{u,\mathrm{tw}}{/t}_{w}}{V_{u,2.0}/2.0\;\mathrm{mm}}$
	[mm]	[kN]	[kN/mm]	[-]
34	2.0	101.77	50.89	1.00
27	2.5	133.04	53.22	1.05
6	3.0	163.07	54.36	1.07

**Table 7:** Comparison of analysis results for girders with a corrugation length of *q* = 155 mm.

No.	*q*	*λ_cL_*	*χ_c_*	*V*_pl,R_	*V*_Rd,EC3_	*V*_u,FE_	*χ*_FE_	*V*_u,FE_/*V*_Rd,EC3_
	[mm]	[-]	[-]	[kN]	[kN]	[kN]	[-]	[-]
1	155	0.32	0.94	166.47	156.69	187.93	1.13	1.20
8		0.31	0.95	166.47	158.27	188.60	1.13	1.19
15		0.29	0.96	166.47	160.28	189.18	1.14	1.18
22		0.35	0.92	249.71	229.36	277.89	1.11	1.21
29		0.37	0.91	332.94	301.32	354.30	1.06	1.18
36		0.37	0.91	138.73	125.77	150.20	1.08	1.19
43		0.43	0.86	110.98	95.79	115.36	1.04	1.20
Average								1.194
STD								0.013

**Table 8:** Comparison of analysis results for girders with a corrugation length of *q* = 232 mm.

No.	*q*	*λ_cL_*	*χ_c_*	*V*_pl,R_	*V*_Rd,EC3_	*V*_u,FE_	*χ_FE_*	*V*_u,FE_/*V*_Rd,EC3_
	[mm]	[-]	[-]	[kN]	[kN]	[kN]	[-]	[-]
2	232	0.41	0.88	166.47	146.25	178.05	1.07	1.22
9		0.39	0.89	166.47	148.28	179.95	1.08	1.21
16		0.37	0.91	166.47	150.83	180.81	1.09	1.20
23		0.46	0.85	249.71	211.96	261.34	1.05	1.23
30		0.48	0.83	332.94	276.58	342.33	1.03	1.24
37		0.47	0.84	138.73	116.89	141.84	1.02	1.21
44		0.54	0.80	110.98	88.58	111.31	1.00	1.26
Average								1.224
STD								0.019

**Table 9:** Comparison of analysis results for girders with a corrugation length of *q* = 310 mm.

No.	*q*	*λ_cL_*	*χ_c_*	*V*_pl,R_	*V*_Rd,EC3_	*V*_u,FE_	*χ_FE_*	*V*_u,FE_/*V*_Rd,EC3_
	[mm]	[-]	[-]	[kN]	[kN]	[kN]	[-]	[-]
3	310	0.49	0.83	166.47	137.86	168.70	1.01	1.22
10		0.47	0.84	166.47	140.22	170.10	1.02	1.21
17		0.44	0.86	166.47	143.19	170.83	1.03	1.19
24		0.55	0.79	249.71	197.99	251.64	1.01	1.27
31		0.59	0.77	332.94	256.70	334.05	1.00	1.30
38		0.55	0.79	138.73	109.84	139.00	1.00	1.27
45		0.64	0.75	110.98	82.95	110.28	0.99	1.33
Average								1.257
STD								0.049

**Table 10:** Comparison of analysis results for girders with a corrugation length of *q* = 388 mm.

No.	*q*	*λ_cL_*	*χ_c_*	*V*_pl,R_	*V*_Rd,EC3_	*V*_u,FE_	*χ_FE_*	*V*_u,FE_/*V*_Rd,EC3_
	[mm]	[-]	[-]	[kN]	[kN]	[kN]	[-]	[-]
4	388	0.56	0.79	166.47	130.87	166.43	1.00	1.27
11		0.53	0.80	166.47	133.50	167.23	1.00	1.25
18		0.50	0.82	166.47	136.79	167.57	1.01	1.23
25		0.64	0.75	249.71	186.42	247.37	0.99	1.33
32		0.69	0.72	332.94	240.28	330.22	0.99	1.37
39		0.63	0.75	138.73	104.03	136.97	0.99	1.32
46		0.73	0.71	110.98	78.34	107.66	0.97	1.37
Average								1.306
STD								0.058

**Table 11:** Comparison of analysis results for girders with a corrugation length of *q* = 465 mm.

No.	*q*	*λ_cL_*	*χ_c_*	*V*_pl,R_	*V*_Rd,EC3_	*V*_u,FE_	*χ_FE_*	*V*_u,FE_/*V*_Rd,EC3_
	[mm]	[-]	[-]	[kN]	[kN]	[kN]	[-]	[-]
5	465	0.63	0.75	166.47	125.18	164.19	0.99	1.31
12		0.60	0.77	166.47	128.01	164.98	0.99	1.29
19		0.56	0.79	166.47	131.54	165.36	0.99	1.26
26		0.72	0.71	249.71	177.08	241.76	0.97	1.37
33		0.79	0.68	332.94	227.07	326.43	0.98	1.44
40		0.71	0.72	138.73	99.32	134.96	0.97	1.36
47		0.81	0.67	110.98	74.65	103.59	0.93	1.39
Average								1.344
STD								0.062

**Table 12:** Comparison of analysis results for girders with a corrugation length of *q* = 542 mm.

No.	*q*	*λ_cL_*	*χ_c_*	*V*_pl,R_	*V*_Rd,EC3_	*V*_u,FE_	*χ_FE_*	*V*_u,FE_/*V*_Rd,EC3_
	[mm]	[-]	[-]	[kN]	[kN]	[kN]	[-]	[-]
6	542	0.69	0.72	166.47	120.36	163.07	0.98	1.35
13		0.65	0.74	166.47	123.34	163.95	0.98	1.33
20		0.61	0.76	166.47	127.05	164.66	0.99	1.30
27		0.80	0.68	249.71	169.22	238.90	0.96	1.41
34		0.87	0.65	332.94	216.01	314.37	0.94	1.46
41		0.77	0.69	138.73	95.36	133.04	0.96	1.40
48		0.88	0.65	110.98	71.54	101.77	0.92	1.42
Average								1.381
STD								0.056

**Table 13:** Comparison of analysis results for girders with a corrugation length of *q* = 620 mm.

No.	*q*	*λ_cL_*	*χ_c_*	*V*_pl,R_	*V*_Rd,EC3_	*V*_u,FE_	*χ*_FE_	*V*_u,FE_/*V*_Rd,EC3_
	[mm]	[-]	[-]	[kN]	[kN]	[kN]	[-]	[-]
7	620	0.75	0.70	166.47	116.09	162.81	0.98	1.40
14		0.71	0.72	166.47	119.19	162.99	0.98	1.37
21		0.66	0.74	166.47	123.05	163.83	0.98	1.33
28		0.87	0.65	249.71	162.32	234.15	0.94	1.44
35		0.96	0.62	332.94	206.34	301.96	0.91	1.46
42		0.84	0.66	138.73	91.86	132.64	0.96	1.44
49		0.96	0.62	110.98	68.81	98.40	0.89	1.43
Average								1.412
STD								0.047

**Table 14:** Comparison of analysis results with previous studies.

Model No.	*τ_FE_*	*τ_n,SB_*	*τ_n,BA_*	*τ_n,EC_*	*τ_FE_*/*τ_y_*	*τ_n,SB_*/*τ_y_*	*τ_n,BA_*/*τ_y_*	*τ*_n,EC_/*τ*_y_
	[MPa]	[MPa]	[MPa]	[MPa]	[-]	[-]	[-]	[-]
1	250.57	176.14	180.12	208.87	1.13	0.79	0.81	0.94
2	237.40	175.98	180.05	195.10	1.07	0.79	0.81	0.88
3	224.93	175.40	179.82	183.78	1.01	0.79	0.81	0.83
4	221.91	173.74	179.22	174.46	1.00	0.78	0.81	0.79
5	218.92	169.91	178.06	166.92	0.99	0.77	0.80	0.75
6	217.43	162.65	176.10	160.48	0.98	0.73	0.79	0.72
7	217.08	151.26	173.11	154.71	0.98	0.68	0.78	0.70
8	251.47	176.14	180.12	211.09	1.13	0.79	0.81	0.95
9	239.93	175.98	180.06	197.77	1.08	0.79	0.81	0.89
10	226.80	175.40	179.84	186.89	1.02	0.79	0.81	0.84
11	222.97	173.74	179.29	178.01	1.00	0.78	0.81	0.80
12	219.97	169.91	178.23	170.69	0.99	0.77	0.80	0.77
13	218.60	162.65	176.45	164.47	0.98	0.73	0.79	0.74
14	217.32	151.26	173.72	158.93	0.98	0.68	0.78	0.72
15	252.24	176.14	180.12	213.75	1.14	0.79	0.81	0.96
16	241.08	175.98	180.06	201.10	1.09	0.79	0.81	0.91
17	227.77	175.40	179.86	190.89	1.03	0.79	0.81	0.86
18	223.43	173.75	179.37	182.45	1.01	0.78	0.81	0.82
19	220.48	169.91	178.43	175.35	0.99	0.77	0.80	0.79
20	219.55	162.65	176.84	169.36	0.99	0.73	0.80	0.76
21	218.44	151.26	174.43	164.03	0.98	0.68	0.79	074
22	247.01	176.13	180.12	203.98	1.11	0.79	0.81	0.92
23	232.30	175.98	180.04	188.45	1.05	0.79	0.81	0.85
24	223.68	175.40	179.74	176.02	1.01	0.79	0.81	0.79
25	219.88	173.74	178.95	165.81	0.99	0.78	0.81	0.75
26	214.90	169.91	177.33	157.37	0.97	0.77	0.80	0.71
27	212.36	162.65	174.52	150.49	0.96	0.73	0.79	0.68
28	208.13	151.25	170.24	144.28	0.94	0.68	0.77	0.65
29	236.20	176.13	180.12	200.88	1.06	0.79	0.81	0.91
30	228.22	175.97	180.03	184.45	1.03	0.79	0.81	0.83
31	222.70	175.39	179.72	171.13	1.00	0.79	0.81	0.77
32	220.15	173.74	178.83	160.26	0.99	0.78	0.81	0.72
33	217.62	169.90	176.97	151.38	0.98	0.77	0.80	0.68
34	209.58	162.65	173.70	144.05	0.94	0.73	0.78	0.65
35	201.31	151.25	168.67	137.62	0.91	0.68	0.76	0.62
36	240.32	176.06	180.09	201.32	1.08	0.79	0.81	0.91
37	226.94	175.59	179.90	187.11	1.02	0.79	0.81	0.84
38	222.40	173.90	179.27	175.79	1.00	0.78	0.81	0.79
39	219.15	169.29	177.74	166.47	0.99	0.76	0.80	0.75
40	215.94	159.70	174.91	158.93	0.97	0.72	0.79	0.72
41	212.86	144.61	170.58	152.49	0.96	0.65	0.77	0.69
42	212.22	126.02	164.73	146.94	0.96	0.57	0.74	0.66
43	230.72	175.76	179.99	191.55	1.04	0.79	0.81	0.86
44	222.62	174.00	179.32	177.13	1.00	0.78	0.81	0.80
45	220.56	168.09	177.29	165.81	0.99	0.76	0.80	0.75
46	215.32	154.45	172.83	156.71	0.97	0.70	0.78	0.71
47	207.18	133.34	165.87	149.38	0.93	0.60	0.75	0.67
48	203.54	110.23	157.15	143.17	0.92	0.50	0.71	0.65
49	196.80	89.58	147.61	137.62	0.89	0.40	0.67	0.62

## Data Availability

The original contributions presented in this study are included in the article; further inquiries can be directed to the corresponding author.

## Appendix A

The symbols and notations used in this article are shown in Table A1.

**Table A1:** Symbols and notations used in this article.

Symbols/Notations	Significance
*a_f_*	wave amplitude
BS	beam with sinusoidal corrugated web
*b*_f_	flanges width
*D*_x_	plate bending stiffnesses in the plane perpendicular to the wave crest
*D*_y_	plate bending stiffnesses in the plane parallel to the wave crest
*E*	Young’s modulus
*f*_u_	tensile strength
*f*_y_	yield strength
GB	global buckling
*H*	girder height
*h*_s_	wave height
*h*_w_	web height
*L*	girder length
LB	local buckling
*L*_e_	distance from the girder end to the support point
LFS	low-frequency sinusoidal
*L*_m_	central segment length
*L*_s_	support zone segment length
*q*	wavelength
*S*	developed wavelength
*t*_f_	flanges thickness
*t*_w_	web thickness
*v*	Poisson’s ratio
*V*_cr_	elastic shear buckling load
*V*_pl,R_	plastic shear capacity
*V*_Rd_	shear capacity
*V*_u_	ultimate shear load
*ε*_nom_	nominal stress
*ε*_true_	true strain
*ε*_u_	ultimate strain
σ_nom_	nominal strain
*σ*_true_	true stress
*τ*	design strength resistance
*τ*_y_	yield strength
*χ*_c_	shear buckling reduction factor
*χ*_cG_	global instability factors
*χ*_cL_	local instability factors
